# Supplementary material for: Prevalence of visual impairment and associated factors among children in Ethiopia: Systematic review and meta-analysis
Source: PLoS One. 2022 Jul 21;17(7):e0271433. doi: 10.1371/journal.pone.0271433 (PMC9302764; doi:10.1371/journal.pone.0271433)
Supplement: Attachment — Submitted filename: Response to reviewers comments.docx [file pone.0271433.s004.docx]

**A rebuttal letter**

Journal name: PLOS ONE

PONE-D-21-27729

**Title: Prevalence of visual impairment and associated factors among children in Ethiopia: Systematic review and meta-analysis**

The Editor’s, reviewers’ comments, and point by point response of the authors are presented by the following table. For the sake of presenting the response clearly, we preferred the table form for this response.

| **Editor’s comments** | **Authors’ response** |
| --- | --- |
| 1. Please ensure that your manuscript meets PLOS ONE's style requirements, including those for file naming. The PLOS ONE style templates can be found at  <https://journals.plos.org/plosone/s/file?id=wjVg/PLOSOne_formatting_sample_main_body.pdf> and  https://journals.plos.org/plosone/s/file?id=ba62/PLOSOne_formatting_sample_title_authors_affiliations.pdf | Thank you editor, for your valuable comment. As you commented us, we prepared the manuscript as per the PLOS ONE's style requirements. |
| 2. Please provide the full electronic search strategy for at least one database, including any limits used, such that it could be repeated | Thank you Editor, for you comment. As you commented us, the full electronic search strategy is included in the method part of the manuscript under the heading “Design and searching strategy”, using the appropriate and inclusive combinations. |
| 3. We note that you have indicated that data from this study are available upon request. PLOS only allows data to be available upon request if there are legal or ethical restrictions on sharing data publicly;  b. If there are no restrictions, please upload the minimal anonymized data set necessary to replicate your study findings as either Supporting Information files or to a stable, public repository and provide us with the relevant URLs, DOIs, or accession numbers | Thank you editor, for your priceless comments. We need to be clear about the data availability issue. Even though we said that data from this study are available upon request, there is no any legal or ethical restrictions with it. As you requested us, therefore, the minimal anonymized data set necessary to replicate our study findings is uploaded as Supporting Information file. |
| 4. Please include captions for your Supporting Information files at the end of your manuscript, and update any in-text citations to match accordingly. Please see our Supporting Information guidelines for more information: <http://journals.plos.org/plosone/s/supporting-information>. | Thank you editor, for offering us your valuable comments. Unfortunately, we have no any supporting file to be published as part of the manuscript. The dataset, however, is uploaded as supporting information file for editor only. Hence, it doesn’t require any caption or citation in the manuscript. |
| 5.  We noticed you have some minor occurrence of overlapping text with the following previous publication(s), which needs to be addressed:  - <https://www.hindawi.com/journals/joph/2020/6934013/> - <https://bjo.bmj.com/content/bjophthalmol/97/7/812.full.pdf> - <https://bmcinfectdis.biomedcentral.com/articles/10.1186/s12879-019-4686-8>  In your revision ensure you cite all your sources (including your own works), and quote or rephrase any duplicated text outside the methods section. | We would like to give you our gratitude, for your kind and valuable comments. As per your comment and request, we made the necessary correction in the revised manuscript by citing all the necessary articles. Ideas which appear similar with others are paraphrased properly. |
| While revising your submission, please upload your figure files to the Preflight Analysis and Conversion Engine (PACE) digital diagnostic tool, <https://pacev2.apexcovantage.com/>. | Thank you Editor for your valuable guidance. We have submitted all figures with the updated manuscript, as requested, after uploading them to the PACE digital diagnostic system. |
| **Comments from Reviewer #1** |  |
| 1. The purpose of this systematic review is to report pooled estimate of the prevalence and associated factors of visual impairment among children in Ethiopia. However the “Determinants of Visual impairment “it covered were not of interest. As the author had discussed in the article, the cause of visual impairment would be of more clinical interest and value. | Thank you reviewer, for your interesting and deep comment. The associated factors included in this systematic review and meta-analysis are those identified by the articles included in this study. The presence or absence of statistical association between the list of factors and the outcome is a finding by itself. It can still point something valuable to the concerned bodies in the area. |
| 2. Literature Searches and Search terms are incomplete. This is suboptimal for publication for systematic review. Search terms should better include any population based study on vision, not only for child. Those studies would include a group of patients in this age range. | Thank you reviewer for your valuable comment. The study was supposed to include both institutional and community based studies, and hence there was no any exclusion made in searching strategy. The searching of literatures was made systematically using very inclusive terms and available MESH terms with appropriate combination. |
| 3. There is substantive heterogeneity in estimating pooled effect size of factors of visual impairment among children. Please explain the way of management in the method, result and discussion. | Thank you reviewer for this constructive comment. However, the p-value for the identified I^2^ values in estimating pooled effect size of factors were found to be insignificant for all factors. Hence, we decided that there is no significant heterogeneity. As you have suggested us, we included this justification in the pointed portions of the revised manuscript. |
| 4. What is the exact criteria for low vision? In the outcome measurement, line 173 less than 1/18, line 177 less than 6/12. Also some writing mistakes like in line 107 met analysis. | Thank you dear reviewer for this important question. The different measurements found indicate different outcomes. Outcomes defined were “low vision”, “blindness”, and “visual impairment”. The outcome of interest for this study is “visual impairment” which can include both low vision and blindness. It is more inclusive than the two aforementioned outcomes. Hence, the last visual acuity, 6/12, is the one which defines visual impairment. Other respective values define low vision and blindness in that order.  Besides, the spelling error you identified, “met analysis”, is corrected in the revised manuscript. |
| **Comments from Reviewer #2** |  |
| **1.TitlePage:** The title is appropriate. The name of institution should be written in capital letter (page 1 line 5 and line 11). | Thank you dear reviewer for your comment. As you commented us the necessary correction is made in the revised manuscript. |
| 2.Introduction The introduction of the study is appropriate. Need to rephrase sentence in line 91 and line92(page3). Paragraph 3 (page 4) is not clear. Need to rephrase/require English editing. Should delete the abbreviation VI (for visual impairment) (page 4 line 95) and BL (for blindness) (page 4 line 97) since those abbreviation are not consistently used in thetext. | Thank you reviewer for your valuable comments. As per your comments, sentences in the indicated lines are paraphrased and abbreviations not consistently used are deleted in the revised manuscript. |
| 3.Methods The methods give enough detail. The name of journal should be written in capital letter (page 5 line 137 and line 138). Need to rephrase sentence in line 142 (page5). Should delete the abbreviation VA (for visual acuity) (page 6 line 177) since this abbreviation is not consistently used in thetext. | Thank you reviewer for your valuable and kind comments. As you commented us, the necessary corrections are made in the revised manuscript. |
| 4.Results The results are presented in a clear and concise manner. | We would like to give our gratitude for your encouraging comments. They are fuels for our motivation. |
| 5.Discussion The discussion interprets the findings based on the results obtained and compare with previous studies for the prevalence of visual impairment. However, for the associated factors for visual impairment, there is lacking comparison with other studies. | Thank you for your constructive comment. We have tried to discuss the factors identified with other studies available. The small number of articles found, however, limits us to discuss it exhaustively. |
| 6.Conclusion The conclusions are valid and based on the results of the study. | We thank you for your time and commitment to evaluate our paper. The respective comments you offered us build us! |
