# Supplementary material for: Prevalence of visual impairment and associated factors among children in Ethiopia: Systematic review and meta-analysis
Source: PLoS One. 2022 Jul 21;17(7):e0271433. doi: 10.1371/journal.pone.0271433 (PMC9302764; doi:10.1371/journal.pone.0271433)
Supplement: Attachment — Submitted filename: Response to reviewers comments 2.docx [file pone.0271433.s005.docx]

**A rebuttal letter 2**

Journal name: PLOS ONE

PONE-D-21-27729

**Title: Prevalence of visual impairment and associated factors among children in Ethiopia: Systematic review and meta-analysis**

Thank you all for giving us your valuable time. The Editor’s, reviewers’ comments, and point by point response of the authors are presented by the following table.

| **Comments from reviewer #1** | **Authors’ response** |
| --- | --- |
| 1. The study was supposed to include both institutional and community based studies, and hence there was no any exclusion made in searching strategy. My concern is there might be other population based studies which include child. The searching combination " child [Title/Abstract] OR children [Title/Abstract] OR childhood [Title/Abstract] OR "school age children"[Title/Abstract] might exclude these studies. | Thank you for your comment. As we have said before, there was no any exclusion made in the searching strategy regarding to institutions where the studies might be conducted. Therefore, there was no way that community based studies on visual impairment among children would be missed. |
| 2. My understanding from table 2, all the I2 indexes were larger than 30%. It indicates the variability in the measured effect sizes across studies is caused by true heterogeneity among studies. Though a non-significant value of Q statistics (not I2) suggests that the studies are homogeneous, the Q statistic has limited power to detect heterogeneity in meta-analyses with few studies (ie 2-6 in this study). | Thank you reviewer for your valuable comment. The insignificant p-values of Q statistics assured us the absence of true heterogeneity in the effect sizes even though it has limited power. We preferred not to use random effect model even though the I statistics is elevated, for it is not advisable to use it when studies are small. |
| 3. Outcomes defined were “low vision”, “blindness”, and “visual impairment”. The outcome of interest for this study is “visual impairment” which can include both low vision and blindness. It is more inclusive than the two aforementioned outcomes. Hence, the last visual acuity, 6/12, is the one which defines visual impairment. Other respective values define low vision and blindness in that order. This is hard to understand. If visual impairment includes both low vision and blindness, why 6/12 not 6/18? | Thank you for this important comment. Something which has to be clear here is that our outcome of interest is “visual impairment” of any extent. We can forget about “low vision” and “Blindness”. The maximum visual acuity measurement value which defines visual impairment is 6/12. All results of visual acuity measurement bellow 6/12 will also indicate the presence of visual impairment. As you have said, visual acuity measurement 6/18 also indicates visual impairment, but it is not the maximum limit which defines the outcome. |
| **Comment from Reviewer #2:**  The authors have adequately addressed my comments. | Thank you dear reviewer, for giving us your valuable time for the improvement of our manuscript. |
