# Supplementary material for: Prevalence of visual impairment and associated factors among children in Ethiopia: Systematic review and meta-analysis
Source: PLoS One. 2022 Jul 21;17(7):e0271433. doi: 10.1371/journal.pone.0271433 (PMC9302764; doi:10.1371/journal.pone.0271433)
Supplement: Attachment — Submitted filename: Response to editors and reviewers comments 3.docx [file pone.0271433.s006.docx]

**A rebuttal letter 3**

Journal name: PLOS ONE

PONE-D-21-27729

**Title: Prevalence of visual impairment and associated factors among children in Ethiopia: Systematic review and meta-analysis**

Thank you for giving us your valuable time. The Editor’s comments and point by point responses of the authors are presented by the following table.

| **Comments from the editor** | **Authors’ response** |
| --- | --- |
| 1. Thank you for your responses to Reviewer 1. I suggest that you make a few minor edits in your manuscript so that readers not from the eye field can understand the subject matter better. For example: a. You can include a line in your Methods, highlighting that "the search strategy included both institutional and community-based studies". The aim is to ensure your readers understand that the robustness of your search strategy. b. Also in Methods, highlight that "we have chosen X, instead of random effect model, even though the I statistics is elevated because the studies are small (cite)." The aim is to ensure your readers understand the rationale of your choice of analysis. c. Also in Methods, highlight that "we defined vision impairment as vision worse than 6/12 in the better eye as it includes low vision (VA definition) and blindness (VA definition)". | Thank you for your valuable comments. As per your comment, we have highlighted each responses mentioned “a to c” in the methods and materials section of the revised manuscript. The changes made are also indicated in the revised manuscript with track changes. |
| 2. Please review your reference list to ensure that it is complete and correct. If you have cited papers that have been retracted, please include the rationale for doing so in the manuscript text, or remove these references and replace them with relevant current references. Any changes to the reference list should be mentioned in the rebuttal letter that accompanies your revised manuscript. If you need to cite a retracted article, indicate the article’s retracted status in the References list and also include a citation and full reference for the retraction notice. | Thank you for your valuable comment. As per your recommendation, we have reviewed the references for completeness and correctness. We found that all are correct and complete. No retracted papers are cited. |
