# Supplementary material for: Prevalence of visual impairment and associated factors among children in Ethiopia: Systematic review and meta-analysis
Source: PLoS One. 2022 Jul 21;17(7):e0271433. doi: 10.1371/journal.pone.0271433 (PMC9302764; doi:10.1371/journal.pone.0271433)
Supplement: Attachment — Submitted filename: Reviewer comments.docx [file pone.0271433.s003.docx]

General

The article ‘Prevalence of visual impairment and associated factors among children in Ethiopia:

Systematic review and meta-analysis’ is very interesting and provide useful information to readers.

The manuscript contains original findings.

However, there are few issues need to be consider.

Title Page

The title is appropriate.

Th name of institution should be written in capital letter (page 1 line 5 and line 11).

Abstract

The abstract summarizes clearly and concisely the main finding of the results.

Main Manuscript

1. Introduction

The introduction of the study is appropriate.

Need to rephrase sentence in line 91 and line 92 (page 3).

Paragraph 3 (page 4) is not clear. Need to rephrase/require English editing.

Should delete the abbreviation VI (for visual impairment) (page 4 line 95) and BL (for blindness) (page 4 line 97) since those abbreviation are not consistently used in the text.

2. Methods

The methods give enough detail.

The name of journal should be written in capital letter (page 5 line 137 and line 138).

Need to rephrase sentence in line 142 (page 5).

Should delete the abbreviation VA (for visual acuity) (page 6 line 177) since this abbreviation is not consistently used in the text.

3. Results

The results are presented in a clear and concise manner.

4. Discussion

The discussion interprets the findings based on the results obtained and compare with previous studies for the prevalence of visual impairment.

However, for the associated factors for visual impairment, there is lacking comparison with other studies.

5. Conclusion

The conclusions are valid and based on the results of the study.

References

There are missing name of journal in few number of references.

Figures

The figures and figure legend are appropriate, clear and corretly labelled.

Tables

The tables and table legend are appropriate, clear and corretly labelled.
